# The Impact of LED Color Rendering on the Dark Adaptation of Human Eyes at Tunnel Entrances

**DOI:** 10.3390/ijerph17051566

**Published:** 2020-02-28

**Authors:** En-Zhong Zhao, Li-Li Dong, Yang Chen, Qi Lou, Wen-Hai Xu

**Affiliations:** School of Information Science and Technology, Dalian Maritime University, Dalian 116026, China; zhaoenzhong@dlmu.edu.cn (E.-Z.Z.); chenyang123@dlmu.edu.cn (Y.C.); louqi@dlmu.edu.cn (Q.L.); xuwenhai@dlmu.edu.cn (W.-H.X.)

**Keywords:** dark adaptation, color rendering, tunnel entrance, traffic safety, tunnel lighting

## Abstract

The dark adaptation of drivers’ eyes at a tunnel entrance seriously affects traffic safety. This can be improved by the design of tunnel lighting. Light-Emitting Diode (LEDs) have been applied as a new type of luminaire in tunnel lighting in recent years, but at present, there are few studies on the influence of color rendering of LEDs on tunnel traffic safety, and there is no explicit indicator for the selection of appropriate color rendering parameters in tunnel lighting specifications, which has aroused researchers’ concern. In this article, several new color rendering evaluation indexes were compared, and as a result, it is considered that CRI2012 (a color difference-based color rendering index) is more suitable for evaluating the color rendering of LEDs used at tunnel entrances. The dark adaptation phenomenon was simulated in the laboratory. Four CRI2012s, three color temperatures and eight colored targets were used in the experiments. The results showed that yellow, silver and white can provide shorter reaction times, while red and brown lead to longer reaction times, which can provide a reference for the design of road and warning signs at tunnel entrances. The effect of target color on reaction time was greater than that of color rendering. Under most target colors, the higher the CRI2012, the shorter the reaction time. When designing the color rendering of the LEDs at a tunnel entrance, the value should thus be as large as possible (close to 100), and a lower color temperature value (about 2800 K) should be selected. This paper provides technical support for tunnel lighting design and a reference for tunnel lighting specifications, which is of significance to improve driving safety and avoid traffic accidents in highway tunnels.

## 1. Introduction

Highway tunnel entrances are the sections with the highest traffic accident rate in the whole tunnel [1,2,3]. Although fewer accidents occur in tunnels than on open roads [4,5,6], the casualties and losses of traffic accidents happening in tunnels are more serious than those on open roads [7,8,9]. The main factor that causes the frequent traffic accidents at the tunnel entrance is the dark adaptation of human eyes. The drivers’ dynamic visual characteristics are most closely related to traffic accidents [10,11]. Traffic accidents will bring serious threats to personal safety and property safety. As a result, it is very significant to analyze the causes of traffic accidents and improve the traffic environment at tunnel entrances.

When a driver enters a tunnel during the daytime, the human eye will suffer a “black hole effect” due to the sharp change in luminance [12], which will become more obvious when the difference between the internal and external luminance is large [13,14,15]. As the driving speed on highways is relatively fast, serious traffic accidents may occur if the reaction time is too long [16,17]. Therefore, reducing the dark adaptation period can increase the traffic safety factor at the tunnel entrance.

In addition to limiting the vehicle speed at the tunnel entrance, the effect of dark adaptation is usually attenuated by reducing the luminance difference between inside and outside of the tunnel. Limited by the traditional luminaires such as high pressure sodium lamps, whose characteristic parameters like correlated color temperature (CCT) and color rendering are fixed, tunnel lighting was designed based on luminance [18,19,20]. Light-Emitting Diodes (LEDs), which have the advantages of low light attenuation, high luminous efficiency, long life and energy savings, have been widely used in tunnel lighting in recent years. As the parameters of LEDs including CCT and color rendering are not fixed, the applicability and rationality of these parameters in tunnel lighting are not clearly considered in the current specifications. As a result, researchers have begun to focus on the effect of characteristics of LEDs on tunnel lighting. Studies have shown that three important characteristics of LED—CCT, color rendering, and luminous intensity—all play significant roles in driving safety [21,22,23]. At present, there are many studies on the influence of luminance and CCT on tunnel driving safety [21,24,25,26], but few on the influence of color rendering on tunnel driving safety.

There are some studies on the application of color rendering in tunnel lighting and open roads. In 2009, Ekrias [27] and others stated that in road lighting environments colors have a major effect on target visibility in road lighting with lamps of adequate color rendering properties. It is not known whether the use of light sources with good color rendering properties can actually reduce traffic accident rates by improving the visibility of colored targets. In 2016, Deng [28] and others studied the effect of tunnel light color to drivers’ visual performance. Two tunnels were selected to carry out a small target identification experiment. The results showed that the higher the color rendering, the better drivers can identify the obstacle. In 2017, Zhang [29] and others used 15 light combinations (five CCTs and four color rendering indexes (CRIs) incomplete traversal combinations) to study the effect of color rendering on visual performance in tunnel. The results showed that increasing the light color rendering can improve the visibility without increasing the light power. These studies offer few selections for color rendering parameters. Although they chose different color rendering values with different CCTs, they did not control the consistency of the CCT of the lights used in experiments at different color rendering indices. Besides, not all luminaries are LEDs and their spectra are not the same as that in tunnels. To make the results more convincing, in this paper, for the selection of parameters, CCT is kept at the same value while different color rendering properties are selected, and a variety of common CCTs are considered in order to maintain the accuracy and comprehensiveness of the experimental results. As for the selection of spectrum of LEDs, it is consistent with the LED spectrum commonly used in tunnels, both of which are bimodal discontinuous spectra to make the conclusions easier to apply to practice.

While are few studies on the application of color rendering in tunnel lighting, there are more in museums [30], homes and office lighting scenarios [31]. These studies generally agree that LEDs with high color rendering are more suitable for indoor lighting. The experiments in these studies are generally based on subjective feelings, while the research in this paper is based on an objective parameter—reaction time. In addition, there are some studies on the effect of color rendering on visual properties. In 2003, Chee et al. [32] studied visual acuity with different CCT and color rendering of light sources. The results showed that visual acuity is approximately proportional to the average color rendering index under fluorescent lamps, high pressure sodium lamps, metal halide lamps and electrodeless discharge lamps. In 2015, Watanuki [33] found that the color rendering property certainly affects color emotions. Males and females feel different color emotions from skin color. Males feel “lightness” of color while females feel “activity” of color first. The lightness factor shows a correlation with the intensity of illumination, and the activity factor has a negative correlation with the intensity of illumination and alternative CRI. In 2017, Huang et al. [34] conducted a series of psychophysical experiments to investigate and compare the effect of certain factors on color preference, including spectral power distribution (SPD) of light, lighting application, observers personal color preference, regional cultural difference and gender difference. The results showed that the impact of SPD on color preference is significantly stronger than that of other factors, as well as their interactions. Although these studies did not involve specific application scenarios, they can also provide references for the research in this paper. These studies prove that color rendering does affect some visual characteristics, but few studies have linked color rendering to tunnel traffic safety and there is little research on the effect of color rendering on dark adaptation in tunnel entrances. In this article, the effect of color rendering on reaction time is mainly studied, which is directly related to personal safety.

Generally speaking, most studies on color rendering are based on subjective perception, and these studies generally show a positive correlation between color rendering and visual characteristics. In the past few years, our laboratory has provided lighting design recommendations for several tunnels of the Heda highway in Jilin Province of China and accumulated some practical engineering experience and issues to be improved. The tunnel lighting design department and management pay great attention to the selection of color rendering, but there are no official guidelines for reference. The specifications for tunnel lighting [35] do not yet have provisions on which color rendering LEDs are better. Therefore, it is of great significance to study the effect of different color rendering LEDs on tunnel lighting.

**Figure 1:**
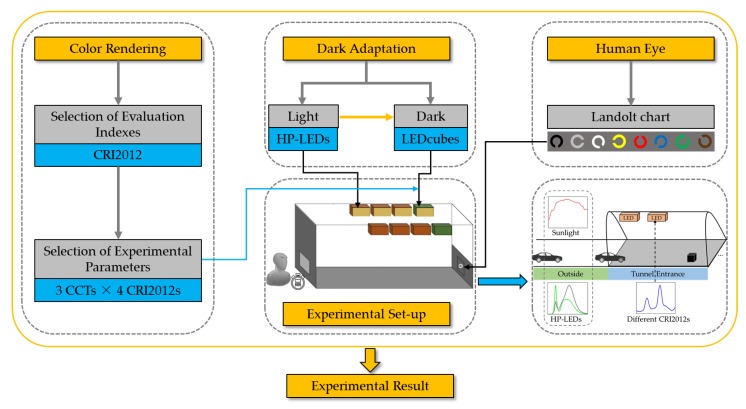
Overview of each part of the article. CRI2012 indicates a color difference-based color rendering index; CCT = correlated color temperature; LED = Light-Emitting Diodes; HP-LEDs = high power LEDs; LEDcube indicate a kind of LED lamp.

Figure 1 shows an overview of several important aspects of this article. Firstly, a more suitable color rendering valuation index in this research was discussed and selected. Four CRI2012s (a color difference-based color rendering index) with three different CCTs were designed to simulate the lighting environment at a tunnel entrance. Secondly, the dark adaptation was simulated by designing a dynamic reduction in luminance based on the luminance outside and inside the tunnel. Thirdly, a Landolt chart was designed using the common car colors for observation. The experiment was conducted in the laboratory simulating the tunnel environment and the degree of dark adaptation is indicated by the reaction time of the subjects.

The purpose of this paper is to investigate the effect of color rendering on dark adaptation at tunnel entrances, which has been rarely studied so far. The results showed that high CRI2012 can improve the dark adaptation at the tunnel entrance and reduce the reaction time of drivers. LEDs with high CRI2012 are recommended for tunnel entrances. By improving the dark adaptation and reducing their reaction time, drivers can identify the obstacles ahead faster. This paper provides technical support for tunnel lighting design and a reference for tunnel lighting specifications, which is of significance to improve driving safety and avoid traffic accidents in highway tunnels.

## 2. Materials and Methods

### 2.1. Color Rendering iIndex Evaluation

At present, the evaluation index of color rendering is still controversial. To specify the visual rendering properties of a light source, the Commission Internationale de l’Eclairage (CIE) proposed a method called Color Rendering Index (CRI) [36], which has been improved over the years [37,38]. With the advent of new types of lighting devices like LEDs, CRI cannot provide a reliable measurement [39,40,41], as it fails to evaluate LEDs with discontinuous spectra [42,43]. In view of this problem, many researchers have provided new color rendering evaluation methods considering: Color Quality Scale (CQS) [44], Gamut Area Index (GAI) [45], CRI2012 [46], etc. Some researchers have made a series of comparisons of these methods [47,48,49]. These methods have made some improvements to CRI, but also have some limitations, as they measure the color rendering from different perspectives (color matching, fidelity, quality, preference, memory, etc.). The most widely used luminaires in the tunnel are LED lamps with noncontinuous spectra.

CRI2012 uses a CIE endorsed state-of-the-art color appearance model (CAM02-UCS) and for the calculation of special indices, using uniform sampling of wavelength space to avoid selective optimization—that is, taking advantage of the unequal contributions of different wavelength regions to the general color rendering score—of light source SPDs [50]. Although CQS also performs well among the above metrics, the resemblance of object colors to their appearance under a well-known reference illuminant is critical and optimizing for an increased chroma using CQS could result in misleading color decisions. For most general interior lighting applications, the increase of chroma is generally not desirable. CRI2012 (a color difference-based color rendering index) eliminated the influence of CRI that some measurement results were unreliable due to nonuniform color space. As a result, in most general lighting applications, the CRI2012 will be the most important target parameter. Therefore, we employ CRI2012 in this article to study the color rendering of LEDs used in tunnels.

### 2.2. Subjects

Twenty-five subjects with normal vision (or corrected vision) with driver’s licenses participated in the study, including seven women and 18 men, ranging in age from 30 to 51. They all had normal color vision in terms of the Ishihara test and none of them had night blindness.

### 2.3. Parameters Setting

**Figure 2:**
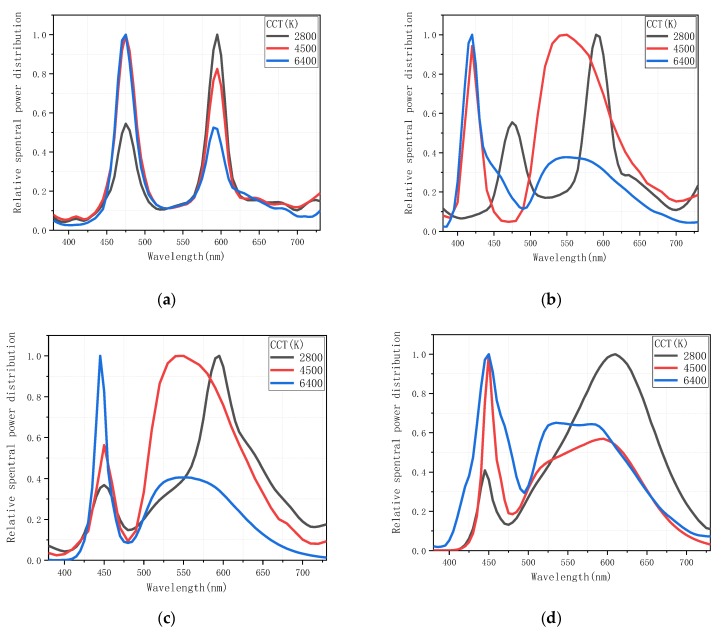
Relative spectral power distribution (SPD) of Light-Emitting Diodes (LEDs) with different CRI2012s (a color difference-based color rendering index) and LP-CCTs (the CCTs simulated by LEDcube). (**a**) CRI2012 = 55 (**b**) CRI2012 = 65 (**c**) CRI2012 = 75 (**d**) CRI2012 = 85.

Two kinds of LEDs were applied in the experiment: LEDcube and high power LED (HP-LED). The LEDcube can simulate different light sources with different characteristics (CCT and color rendering) by input SPD (SPDs are shown in Figure 2 and was used to simulate the light source environment inside the tunnel entrance. HP-LED was used to simulate the sunlight outside the tunnel. In this paper, the CCTs simulated by LEDcube are defined as LP-CCT, and the CCT of HP-LEDs are defined as HP-CCT.

#### 2.3.1. Simulation of CRI2012 and CCT in Tunnel Entrance

The color rendering index of white LEDs used in a tunnel (blue chips to excite yellow phosphors) are generally greater than 55. As a result, four different CRI2012 values were selected (55, 65, 75 and 85, respectively). The difference in color rendering is due to the different SPD values of the LEDs. Different SPDs will influence the CCT value of the LED. CCT can provide people with intuitive color feelings like warm, cold, or yellow, white. Therefore, this article considered the effect of both color rendering and CCT on dark adaptation for more comprehensive consideration. In general, the CCT of white LEDs can cover the range of 2800 K to 6500 K [51,52]. Three different LP-CCTs were selected in the experiment: 2800, 4500 and 6400 K, respectively. Four CRI2012s and three LP-CCTs for a total of 12 lighting conditions in the entrance zone of a tunnel were considered in the experiment.

The SPD curves of different CRI2012s and LP-CCTs are displayed in Figure 2 and were measured by a CS2000 spectroradiometer (Konica Minolta, Tokyo, Japan). All curves follow the dual-peak spectrum LED used in the tunnel. Table 1 shows the specific values of various parameters of 12 experimental luminaries simulating the tunnel lighting, including CCT, deviation of the target from the blackbody locus (duv), CRI2012, CRI and CQS. These parameters were calculated by the software provided by Smet [46] by entering the SPD of the LEDs.

**Figure 3:**
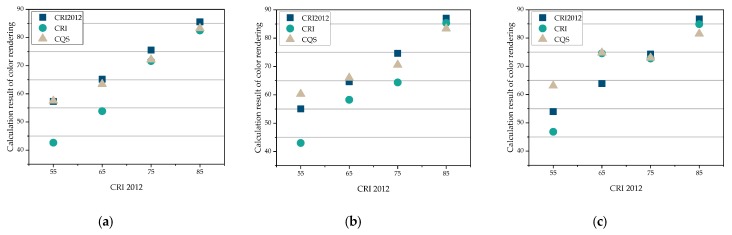
Comparison of three evaluation indexes of color rendering. (**a**) 2800 K. (**b**) 4500 K. (**c**) 6400 K.

Figure 3 shows the comparison of three evaluation indexes of color rendering: CRI2012, CRI and CQS. It can be seen that the curves of CRI2012 and CQS have a higher fitting degree, while the curve of CRI has a lower fitting degree when CCTs are 2800 K and 4500 K.

When CCT is 6400 K, the curves of CRI2012 and CQS are slightly less fitting. In general, when the color rendering performance is better (higher than 85), the three indicators tend to be more consistent.

#### 2.3.2. Simulation of CRI2012 and CCT Outside Tunnel

**Figure 4:**
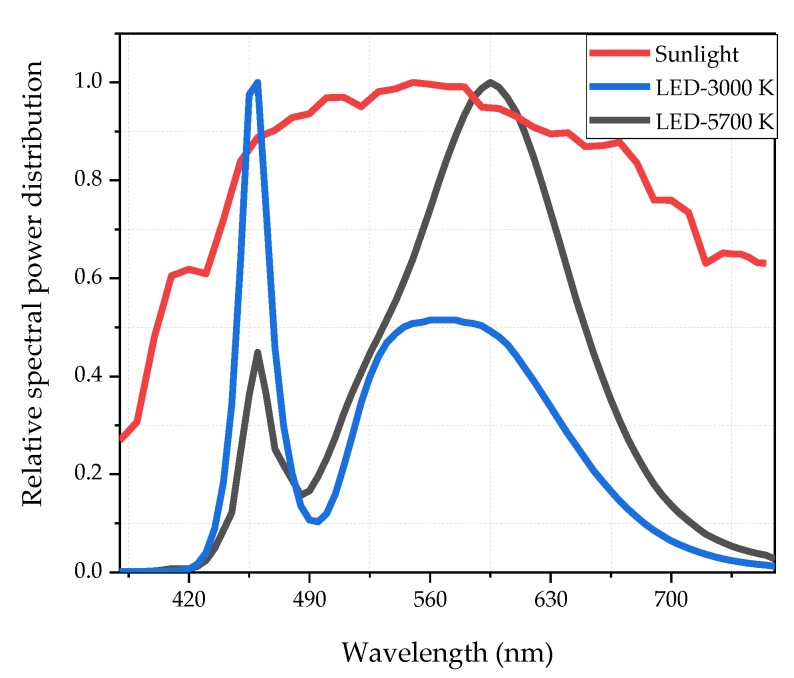
The relative spectral power distributions (SPDs) of 3000 K-LED, 5700 K-LED and sunlight measured at 1 pm.

In 2017, Xiong et al. [53] measured the luminance and CCT of sunlight throughout the day in different months. The results showed that the brightest hours of the day were between 12:00 and 13:00, with CCT ranging from 5000 K to 5700 K. It can be considered that the effect of dark adaptation is most obvious when CCT is in this range. Sunlight cannot be used in the experiment as the luminance as its CCT can’t be controlled, which may affect the results of the experiments. As a result, HP-LEDs with two CCTs—5700 K and 2800 K—were used to simulate the sunlight outside the tunnel. 5700 K, which is usually defined as high CCT, represents the most accident-prone CCT outside the tunnel. 3000 K is compared with the former as a low CCT. The SPDs of HP-LEDs and the sunlight (measured at 1 pm) was shown in Figure 4. The CRI2012 of the sunlight is 99. The CRI2012 of HP-LEDs are 80. Although the SPD values of LEDs do not match the sunlight very well, the luminance and CCT of the HP-LED fits the parameters of the sunlight at midday, when the luminance difference between inside and outside the tunnel is the largest, and when traffic accidents are most likely to occur.

#### 2.3.3. Luminance Value Setting

For the setting of the simulation of the luminance outside the tunnel, in a sunny daytime, the luminance of road surface outside the tunnel can be more than 10000 cd/m^2^. The luminance of simulated road surface using six evenly distributed HP-LEDs in our laboratory is 5000 cd/m^2^, which is lower than that of sunlight in a sunny day at noon, but higher than the annual average for the same period. For the setting of the simulation of the luminance inside the tunnel, according to the 2014 Guidelines [35], the luminance of the entrance zone of a tunnel ranges from 40 cd/m^2^ to 140 cd/m^2^ basing on the designed speed (usually limited to 80 km/h), the traffic volume and other environmental factors [26]. In order to make the experimental effect more obvious, we considered maximizing the difference between internal and external luminance under the condition of matching the actual situation of a tunnel entrance, therefor the luminance selected to simulate the inside of a tunnel was 40 cd/m^2^.

#### 2.3.4. Design of Observation Targets

**Figure 5:**
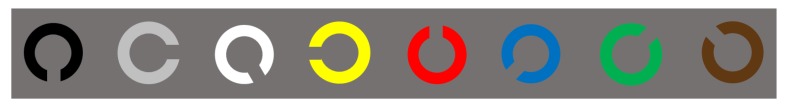
Targets used in experiment with different colors.

A Landolt chart (“C” visual chart) was used in the experiment as the observation target. Its dark adaptation was more obvious than that of the E visual chart in the preliminary experiments. The selection of the targets’ colors refers to the common car colors. In total eight colors were selected in the experiment, as shown in Figure 5: black, silver, white, yellow, red, blue, green, and brown, respectively. The color of the background of targets is similar to that of the tunnel pavement. The orientation of “C” is random, and there are many groups like Figure 5 with random orientations of each color to prevent the subjects from remembering the object orientation and affect the experimental results.

**Figure 6:**
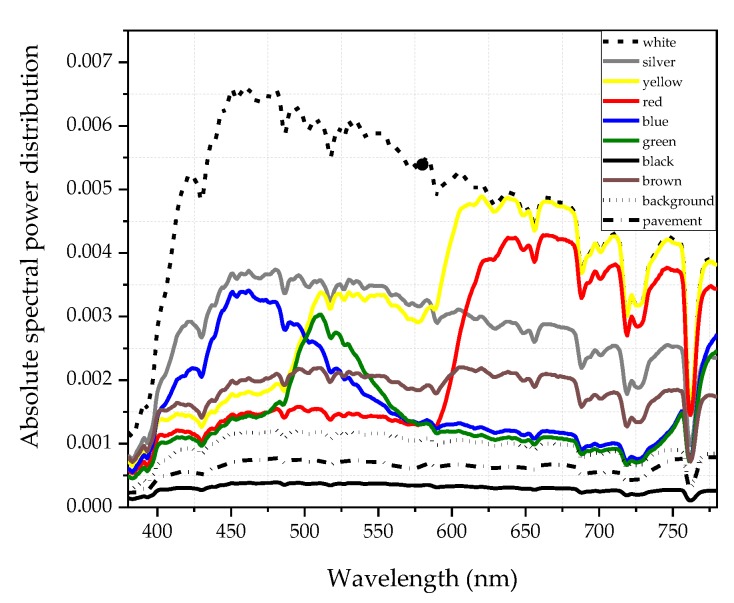
The SPDs of eight targets with different colors, background of targets and tunnel pavement measured by a CS2000 spectroradiometer (Konica Minolta, Tokyo, Japan) in sunlight.

In order to determine the size of the target C, we asked all subjects to observe objects of eight colors in 12 experimental lighting environments in the preliminary experiment. We made sure that everyone could see the orientation of the targets, and made the size as small as possible. The outer diameter of the target C used in the formal experiment finally was 20 mm. Figure 6 shows the SPDs of eight targets with different colors, the SPD of the background of targets and the SPD of tunnel pavement measured by a Konica Minolta CS2000 (Tokyo, Japan, Asia) in sunlight at 1 pm on a sunny day.

### 2.4. Experimental Set-Up

**Figure 7:**
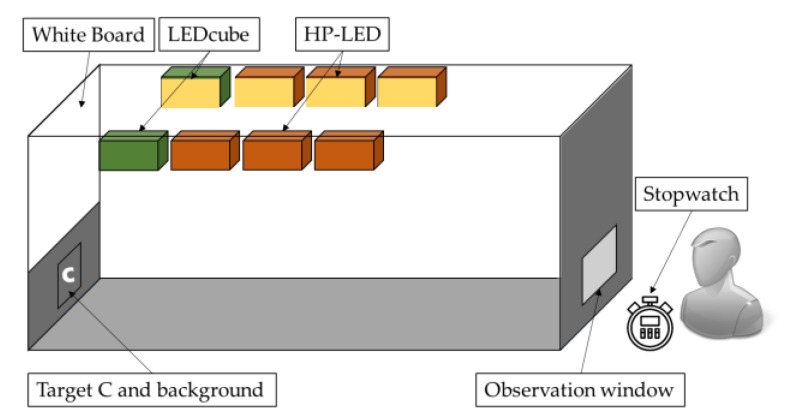
Schematic diagram of experimental set-up. HP-LED refers to the high power LED. Target C refers to the Landolt chart applied in the experiment.

**Figure 8:**
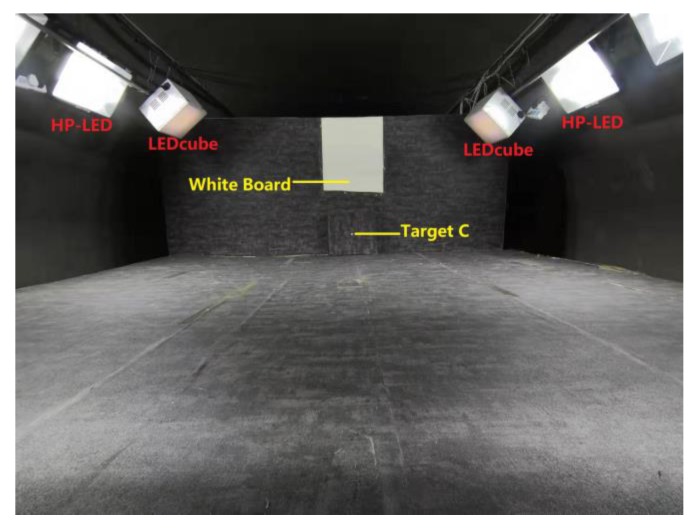
The real experiment situation. Target C refers to the Landolt chart applied in the experiment.

Figure 7 illustrates a schematic diagram of experimental set-up. Figure 8 shows the real experimental situation. Six HP-LEDs and two LEDcubes were suspended on either side of the simulated tunnel. These LEDs were installed 1 m from the horizontal table at a spacing of 0.5 m.

A white board was used for observation by subjects when simulating the environment outside the tunnel. The main purpose of this was to allow the subjects to receive a full lighting spectrum, so as to prevent partial spectrum loss from affecting the experimental results and sufficient luminance can make the experimental results more obvious.

The target “C” with grey background was placed under the white board. The observation window was at the same level as the target, the distance of which was 3 m. The experimental surroundings were painted a concrete color similar to the tunnel walls to simulate the true environment of tunnel as possible. The target “C” was randomly placed in a 1 m^2^ square restricted area (shows in Figure 5, the black border around the target). In each observation experiment, only one target of one color was placed in front of the observer’s line of sight. The orientation of the target was random. The stopwatch was controlled by the observers for timing.

### 2.5. Procedure Specification

Firstly, HP-LEDs and LEDcubes were adjusted to the demanded luminance (5000 cd/m^2^ and 40 cd/m^2^). One kind of CCT of HP-LEDs was selected. LEDcubes were adjusted to a demanded SPD (one kind of CCT and CRI2012) in a random order every time. A target of one color was placed in front of the view. Subjects were asked not to glance the target but to observe the white board through the observation window and take 5 minutes to adjust to ambient brightness.

Secondly, the experimenter turn off HP-LEDs and meanwhile, the subjects detected a change in brightness, pressed the timer in their hand, and looked down at the target area to look for the randomly placed target C. When the subjects perceived the location and then the orientation the target, they pressed the timer again to stop timing. The experimenter recorded the dark adaptation time.

Thirdly, the experimenter changed the color, position and orientation of the target and repeated the procedure above. After eight different colors of objects were tested under the same luminance circumstances, the experimenter adjusted the LEDcubes to another demanded SPD with different CCT or CRI2012. The above process was repeated until all the combinations were tested.

### 2.6. Instruction

To make the results more accurate, each subject underwent three complete experiments, whose results were averaged. In order to familiarize the subjects with the whole experimental process, three preliminary experiments were be conducted in a lighting environment before the formal experiment begins, and these three results are not be considered in the final data. In order to prevent visual fatigue from affecting the results of the experiments, subjects were given a 5-minute rest before the next experiment.

**Figure 9:**
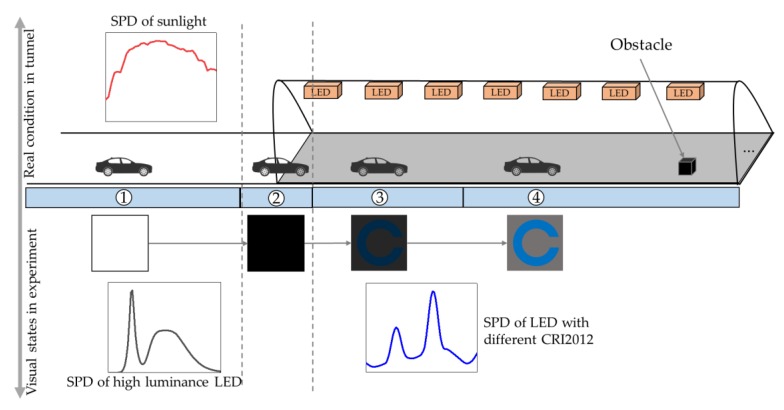
The driving condition of the actual tunnel entrance and the visual states of the subjects in the experiment. SPD = spectral power distribution; LED = light-emitting diode; CRI2012 refers to a color difference-based color rendering index.

Figure 9 shows the driving conditions of the actual tunnel entrance and the visual states of the subjects in the experiment to illustrate the feasibility of the experiment. The whole process of dark adaptation is refined into four states. Firstly, before the driver accesses the entrance of tunnel, the human eyes are exposed to a high luminance. In the experimental environment, HP-LED was used to simulate this high luminance. Secondly, as the driver enters the tunnel entrance, the human eyes experience a sharp reduction in luminance and a sight loss. In the experimental environment, the HP-LED is turned off to achieve this effect. Thirdly, after a period of time, the driver gradually regains his vision and can perceive the presence of obstacles ahead. In the experiment, the subjects could perceive the presence of target C in the designated area after a period of time. Finally, the driver was able to tell the details of the obstacle ahead. In the experiment, after some time, the subjects were able to tell the orientation of the target C.

Although the experimental environment is not completely consistent with a real driving environment, the inevitable errors in a real tunnel, such as changing outdoor luminance, can be avoided in the laboratory. The dynamic process of the tunnel entrance is simulated. The effect of color rendering on reaction time can be obtained accurately by this method.

## 3. Results and Discussion

**Figure 10:**
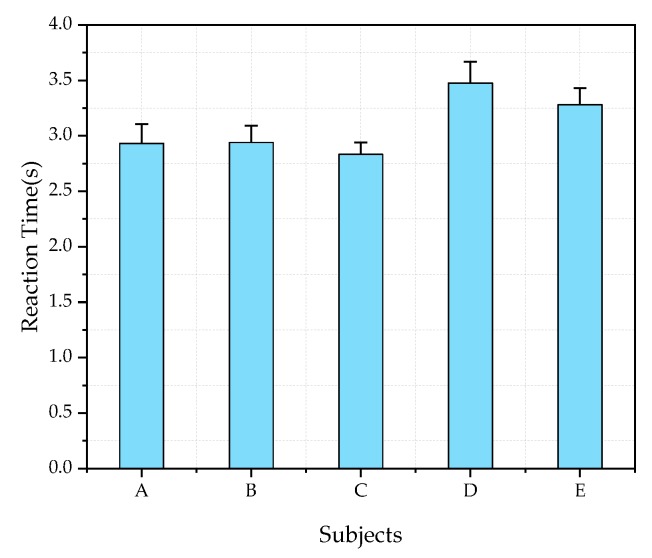
Ten repetitions reaction time of five subjects. CRI2012 = 85, LP-CCT = 2800 K. Error bars indicate the standard deviation.

To measure the error of the experiment, in a preliminary experiment, five of 25 subjects were asked to observe a black target and conduct dark adaptation for 10 times using LEDcubes with CRI2012 of 85 and LP-CCT (the CCTs simulated by LEDcube) of 2800 K as the lighting sources. The HP-CCT is 5700 K. The reaction times of the five subjects are shown in Figure 10. It can be seen that the reaction time value is between 2.5 s and 3.5 s and the value of standard deviation is less than 0.2.

**Figure 11:**
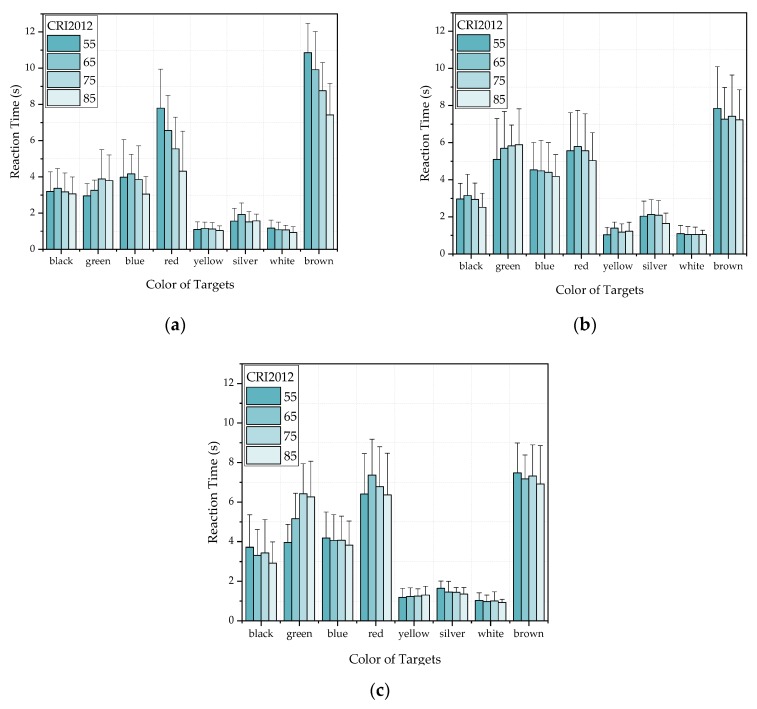
The mean reaction time of all subjects under different CRI2012s (a color difference-based color rendering index) and LP-CCTs (the CCTs simulated by LEDcube) when HP-CCT (the CCT of HP-LEDs) is 5700 K. **(a)** LP-CCT = 2800 K. **(b)** LP-CCT = 4500 K. **(c)** LP-CCT = 6400 K. Error bar shows the standard deviation. CRI2012 refers to a color difference-based color rendering index.

**Figure 12:**
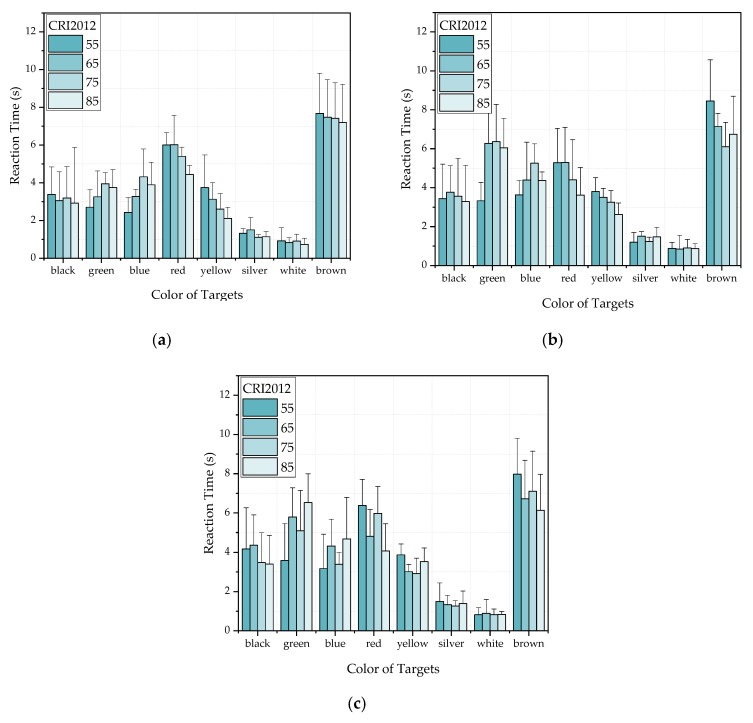
The mean reaction time of all subjects under different CRI2012s and LP-CCTs when HP-CCT is 3000 K. (**a**) LP-CCT = 2800 K. (**b**) LP-CCT = 4500 K. (**c**) LP-CCT = 6400 K. Error bar shows the standard deviation.

Figure 11 and Figure 12 show the mean reaction time of all subjects under different CRI2012s and LP-CCTs under different HP-CCTs. It can be seen that the color of targets greatly affect the reaction time, the CRI2012 and CCT affect the reaction time relatively less. In Figure 11, the experimental data of most colors (black, blue, red, silver, white and brown) showed that the reaction time decreased with the increase of CRI2012. The green one showed that the CRI2012 was positively correlated with the reaction time. The yellow one had no obvious uniform trend for CRI2012 and the reaction time may because that the short reaction time results in an insignificant regularity. In Figure 12, most of the results follow a similar trend to the results in Figure 11 except for the blue target, which showed a positive correlation between color rendering and reaction time. Comparing Figure 11 and Figure 12, their trends were similar and the range of reaction time was close. When HP-CCT = 5700K, the trends of CRI2012 and reaction time were more linear. When HP-CCT = 5700K, LP-CCT = 2800K, the response time difference under different CRI2012s is larger than that under other lighting conditions. It can be seen that the difference of reaction time is less than 1 s under different lighting conditions. However, a difference of 0.1 s in reaction time can greatly increase the probability of traffic accidents [54].

**Figure 13:**
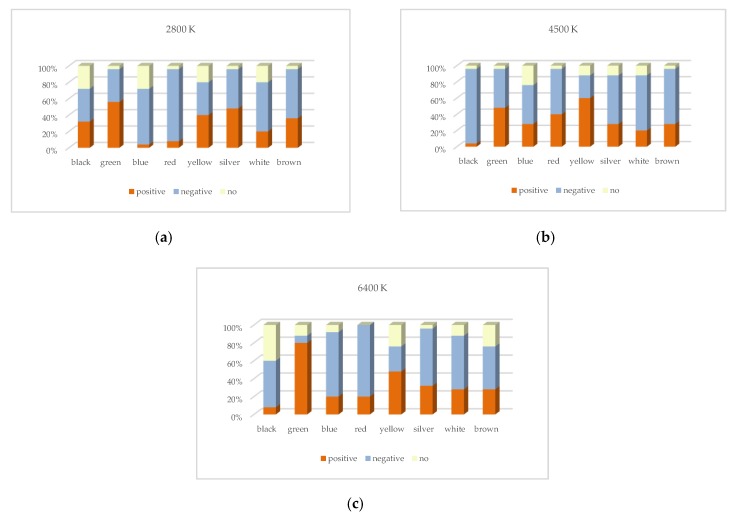
The probability distribution of the correlation between CRI2012 and reaction time when HP-CCT is 5700 K. (**a**) LP-CCT = 2800 K. (**b**) LP-CCT = 4500 K. (**c**) LP-CCT = 6400 K.

**Figure 14:**
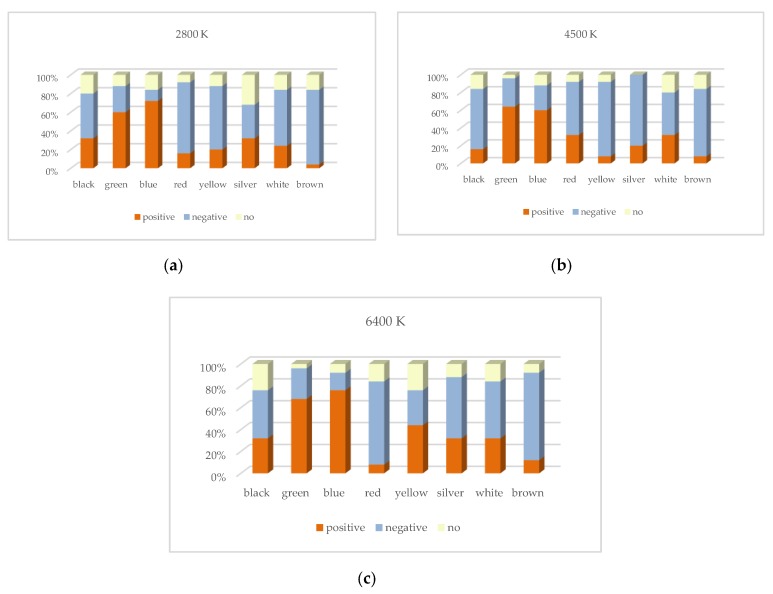
The probability distribution of the correlation between CRI2012 and reaction time when HP-CCT is 3000 K. (**a**) LP-CCT = 2800 K. (**b**) LP-CCT = 4500 K. (**c**) LP-CCT = 6400 K.

**Figure 15:**
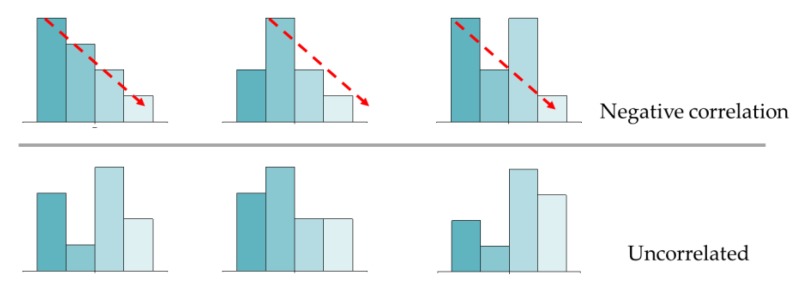
Several possibilities for determining the correlation.

Figure 13 and Figure 14 show the probability distribution of the correlation between CRI2012 and reaction time for 25 subjects observing different colors. For example, when the 25 subjects were asked to observed the black target under LP-CCT = 2800K and HP-CCT = 5700 K, the results of the reaction time showed that among the 25 subjects, eight subjects showed a positive correlation, 10 subjects showed a negative correlation, and seven subjects showed no significant correlation. For the determination of correlation, we judge that if three or four CRI2012s are correlated with reaction time, the CRI2012 are identified as correlated with reaction time. Figure 15 shows several possibilities for determining the correlation. It can be seen in Figure 13 and Figure 14 that the probability of most colors showing a negative correlation is higher, while the probability of showing a positive correlation and no correlation is lower. Although the experimental results of each subject were different, the experimental data of most subjects showed a relatively consistent trend, that is, when observing objects of different colors, the reaction time of most colors decreased with the increase of CRI2012. As a result, it can be concluded that higher CRI2012 can improve the dark adaptation of human eyes and reduce the reaction time.

**Figure 16:**
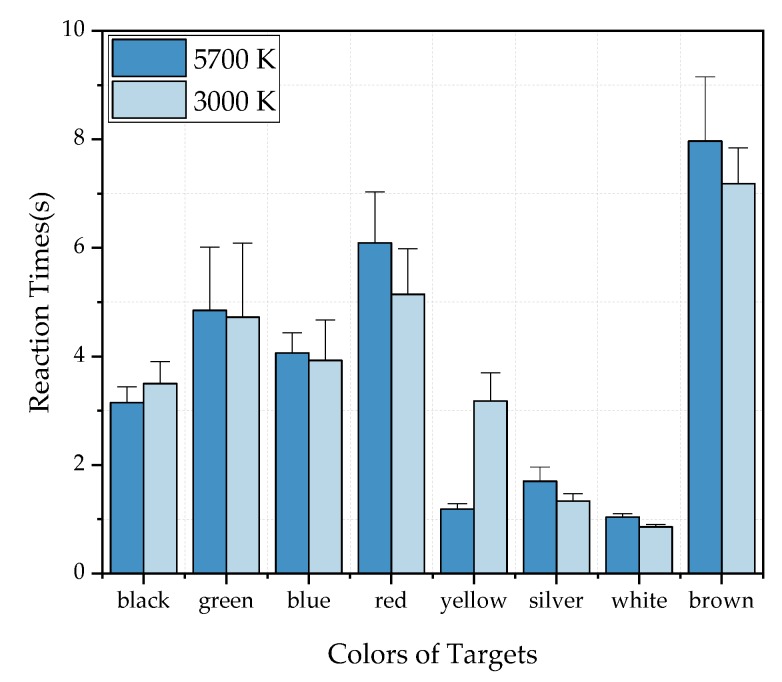
The mean reaction time of different colors under different lighting conditions when HP-CCT = 5700 K and 3000 K. The error bars indicate the standard deviation of different lighting conditions.

The effect of targets with different colors on reaction time is the only consideration in Figure 16. The reaction time of all subjects to observe the same color of targets under different lighting conditions (four CRI2012s and three CCTs) was averaged. It can be seen that, under two HP-LEDs, the two bar charts are similar except for the yellow group. When HP-CCT is 5700 K, the reaction time ranking of different colors from low to high is white, yellow, silver, black, blue, green, red and brown. When HP-CCT is 3000 K, the reaction time of the yellow target is significantly higher. When HP-LED =5700 K, the average reaction time of all colors was longer than that of HP-LED =3000 K, except for the yellow group.

Despite red being intuitively a more striking color, the reaction time of a red target is relatively long in this experiment. Most of the subjects reported in the experimental feedback that when they looked at the red target, they could quickly detect the position and outline of the target, but it was difficult to identify the orientation of the gap in the target. It can be explained by the fact that the contrast between the red target and the cement background is low, which results in the subjects’ discrimination of details being low. The experimental results of this part can provide reference for the color selection of traffic signs at the tunnel entrance. White, yellow and silver are recommended because they are easier to identify by drivers than the other colors. At the same time, cars of these three colors will be more easily perceived by other drivers at the entrance of the tunnel, which should be safer in theory.

**Figure 17:**
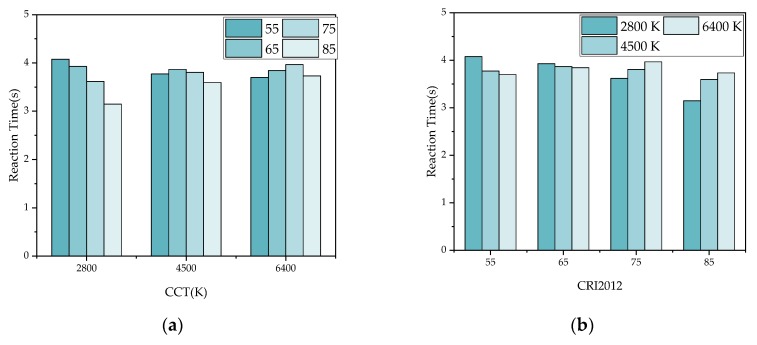
The average reaction time under 12 lighting conditions when HP-CCT is 5700K. (**a**) Take the CCT as the horizontal axis. (**b**) Take the CRI2012 as the horizontal axis

**Figure 18:**
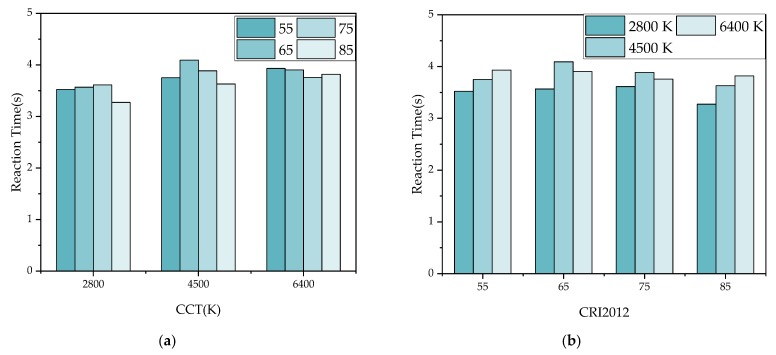
The average reaction time under 12 lighting conditions when HP-CCT is 3000 K. (**a**) Take the CCT as the horizontal axis. (**b**) Take the CRI2012 as the horizontal axis.

Figure 17 and Figure 18 show the reaction time under 12 lighting conditions in Figure 11 and Figure 12. The reaction time of eight colors under each lighting condition is averaged. It can be seen in Figure 17 that when LP-CCT = 2800 K and 4500 K, CRI2012 is negatively correlated with reaction time. When LP-CCT = 6400 K, although CRI2012 is positively correlated with reaction time, the reaction time of high CRI2012 (85) was also very short. In Figure 17b, when CRI2012 = 55 and 65, CCT is negatively correlated with reaction time. When CRI2012 = 75 and 85, CCT is positively correlated with reaction time. In Figure 18, when LP-CCT = 2800 K, CRI2012 is positively correlated with reaction time. When LP-CCT = 4500 K and 6400 K, CRI2012 is negatively correlated with reaction time. It can be seen that the effect of CCT on dark adaptation is inaccurate if only CCT is considered without consideration of CRI2012.

It can be concluded that considering multiple colors, high CRI2012 provides shorter reaction time and improves dark adaptation at tunnel entrance. Under two different HP-CCTs, the reaction time is the shortest when LP-CCT = 3000 K and CRI2012 = 85. Considering both the influence of CCT and CRI2012, LEDs with low CCT (2800 K) and high CRI2012 (over 85) are recommended for the lighting of tunnel entrance. From the experimental results, no significant differences and rules were found in the reaction time data of different genders and ages.

Table 2 shows the significance analysis of the above factors including CRI2012, color, LP-CCT and HP-CCT with reaction time as the dependent variable. It can be seen that the difference of CRI2012, Color, LP-CCT, CRI2012 × Color, Color × LP-CCT and Color × HP-CCT are statistically significant (*p* < 0.05). Color has the most significant effect on reaction time, followed by CRI2012 and LP-CCT, while HP-CCT has no significant effect on reaction time. When studying the effect of LED characteristics on visual characteristics, it is necessary to consider multiple colors as the observation target as they will have a significant impact on the results.

**Figure 19:**
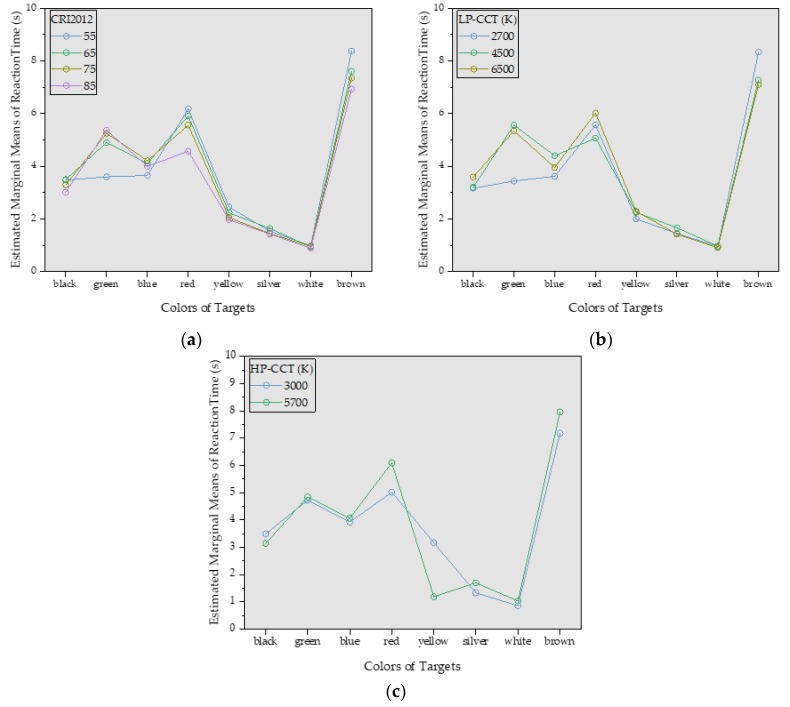
The simple effect analysis of three groups of significant interactions. (**a**) CRI2012 × Color. (**b**) Color × LP-CCT. (**c**) Color × HP-CCT.

Figure 19 shows the simple effect analysis of three groups of significant interactions including (**a**) CRI2012 × Color, (**b**) Color × LP-CCT and (**c**) Color × HP-CCT. In Figure 19a,b, the effect of CRI2012 on reaction time is significantly different when the colors are green, red and brown. In Figure 19c, the effect of CRI2012 on reaction time is significantly different when the colors are red, yellow and brown. Based on the above experimental results, we can condense the following conclusions:

(1) The effect of different colors on reaction time is greater than that of CRI2012 and CCT.
(2) Yellow, silver and white can provide the shortest reaction times, which can provide a reference for the design of road signs and warning signs at tunnel entrances.
(3) For targets of different colors and different CCTs, most subjects had shorter reaction times under high CRI2012, which can lead to the conclusion that LEDs with high CRI2012 are recommended for the lighting design in tunnel entrances. According to the trend of experimental data, it can be inferred that LEDs with CRI2012 value approaching 100 is more suitable for the lighting in tunnel entrance.
(4) For the CCT of LED, under different CRI2012 conditions, the change trend of reaction time with the increase of CCT is not consistent. According to the current experimental results, on the basis of determining the high CRI2012 of LED, the CCT should be selected at a lower value (about 2800 K).

In a previous study [26], we used a method similar to the one described in this article to study the effect of CCT on reaction time at tunnel entrances. However the previous study ignored the interaction of color rendering and CCT on reaction time. This paper is a more comprehensive study on the parameter design of LED at tunnel entrances. Compared with previous studies by other researchers [28,29], this paper considered more kinds of color rendering and CCT, used more colors as the observation targets, and has updated the selection of light sources and evaluation methods for color rendering, which made the results more convincing and obtained conclusions more applicable to actual tunnel entrances.

Although the results presented in the paper can lead to some positive conclusions, there are also some limitations. Firstly, the small sample size may lead to deviation of data regularity and affect the accuracy of the results. Thus, the future studies should increase the case studies sample to ensure a convincing result. Meanwhile, the results will be more accurate by taking into account different characteristics of the samples, such as driving experience and frequency through the tunnel. Secondly, although the experimental environment to the greatest extent represents a real environment, the differences between the two are difficult to assess. Therefore, in the following research, experiments will be conducted in real tunnels. Eye trackers and other devices will be applied to measure reaction times. Comparing further results with the conclusions of this paper may lead to more convincing conclusions.

## 4. Conclusions

In this paper, the effects of color rendering on dark adaptation of human eye in tunnel entrance were analyzed from the point of view of traffic safety. Firstly, the influence of dark adaptation at tunnel entrances on traffic safety is discussed. Color rendering is one of the important characteristics of LEDs, and its research significance to driving safety is discussed. It is explained that the current color rendering evaluation index (CRI) is not applicable to evaluate LEDs. Several new evaluation indexes were compared, and it is considered that CRI2012 is more suitable for evaluating the color rendering of LEDs used at tunnel entrances.

Then, a reaction time experiment was designed to investigate the relationship between CRI2012 and reaction time. In the experiment, four CRI2012s, three CCTs and eight colors of targets were used to simulate in the laboratory the visual dynamic state of a driver at the entrance of a tunnel. In the experiments, the method of switching from HP-LED to low-brightness LED was used to simulate the dark adaptation phenomenon at the tunnel entrance. The similarities and differences between experimental environment and the actual tunnel are discussed. Twenty five subjects with different driving experiences on open road and frequencies through tunnels attended the experiment.

The results showed that the color of targets greatly affects the reaction time, the CRI2012 and CCT affecting the reaction time less, relatively, which can provide a reference for the design of road signs and warning signs at tunnel entrances. High CRI2012 can improve the dark adaptation at the tunnel entrance and reduce the reaction time of drivers. When designing the luminaires at the tunnel entrance, the luminance should be designed considering LEDs with high color rendering. Theoretically, the higher the color rendering of the LEDs, the better for traffic safety. This paper provides a reference for the design of traffic signs, warning signs and vehicle colors at the tunnel entrance combined with color rendering performance. This paper provides a reasonable reference for tunnel lighting specification and tunnel lighting design departments, which can make a contribution to ensuring personal safety and avoiding traffic accidents at tunnel entrances.

## Figures and Tables

**Table 1:** The specific values of various parameters of 12 experimental luminaries simulating the tunnel lighting. LP-CCT refers to the CCT simulated by LEDcube. CRI2012 refers to a color difference-based color rendering index; CCT = correlated color temperature; duv refers to the chromaticity difference from the Planckian or daylight locus; CRI = color rendering index; CQS = Color Quality Scale.

Experiment Parameter		The Calculated Value of Experimental Parameters				
LP-CCT(K)	CRI2012	CCT(K)	duv	CRI2012	CRI	CQS
2800 ± 100	55	2781	−0.02654	57	43	58
	65	2894	−0.01797	65	54	63
	75	2760	–0.00987	76	72	72
	85	2743	+0.00008	86	82	83
4500 ± 100	55	4484	–0.03641	55	43	60
	65	4494	+0.03781	65	58	66
	75	4498	+0.03333	75	64	71
	85	4578	–0.00192	87	85	83
6400 ± 100	55	6425	–0.02966	54	47	63
	65	6379	–0.00425	64	75	75
	75	6442	+0.00478	74	73	73
	85	6444	–0.00160	87	85	81

**Table 2:** P value of significance analysis for the above factors

**CRI2012**	**Color**	**LP-CCT**	**HP-CCT**
0.002	0.000	0.002	0.527
**CRI2012 × Color**	**CRI2012 × LP-CCT**	**CRI2012 × HP-CCT**	**Color × LP-CCT**
0.000	0.244	0.771	0.000
**Color × HP-CCT**	**LP-CCT × HP-CCT**	**CRI2012 × Color × LP-CCT**	**CRI2012 × Color × HP-CCT**
0.000	0.090	0.952	0.206
**CRI2012 × LP-CCT × HP-CCT**	**Color × LP-CCT × HP-CCT**	**CRI2012 × Color × LP-CCT × HP-CCT**	
0.312	0.269	0.394	
